# Case report: Severe cholestatic jaundice associated with hyperthyroidism treated with methimazole

**DOI:** 10.1097/MD.0000000000035972

**Published:** 2023-11-10

**Authors:** Xiaoqiang Liu, Boming Xu, Yilin Zeng, Peizhong Chen, Yubin Wang

**Affiliations:** a Department of Gastroenterology, First Hospital of Quanzhou Affiliated to Fujian Medical University, Quanzhou, Fujian, China.

**Keywords:** cholestatic jaundice, hyperthyroidism, liver dysfunction

## Abstract

**Rationale:**

We present a case of a 43-year-old female patient diagnosed with hyperthyroidism. This study aims to demonstrate the rare association between hyperthyroidism and severe cholestatic jaundice, and the effectiveness of methimazole treatment.

**Patient concerns:**

The patient developed severe jaundice, a typically mild symptom in most hyperthyroidism cases.

**Diagnosis:**

The severe jaundice was suspected to be a result of cholestasis induced by hyperthyroidism, with other potential causes such as drug-induced or autoimmune liver dysfunction being ruled out.

**Outcomes:**

The patient was effectively treated with methimazole. Outcomes: Treatment with methimazole alleviated the severe cholestatic jaundice and restored normal thyroid function.

**Lessons:**

The specific mechanism of cholestasis as a secondary complication of hyperthyroidism remains unclear, and there are no specific biochemical markers for cholestasis caused by this hormonal disease. This case underscores the possibility of severe jaundice as a clinical manifestation of hyperthyroidism, and highlights antithyroid drug treatment as an effective strategy for managing severe cholestatic jaundice.

## 1. Introduction

Hyperthyroidism is a prevalent endocrine disorder characterized by an overproduction and excessive levels of thyroid hormones in the bloodstream. The condition can elicit a series of systemic symptoms including, but not limited to, tachycardia, excessive sweating, weight loss, irritability, and anxiety.^[1]^ Methimazole (MMI), an antithyroid drug, is widely used in the treatment of hyperthyroidism. However, instances of hyperthyroidism complicated by severe cholestatic jaundice are relatively uncommon. Cholestatic jaundice arises from a blockage in bile secretion or excretion, leading to an accumulation of bilirubin in the blood.^[2]^ This condition can result in various complications, such as infections, malnutrition, and a propensity for bleeding, severely impacting the patient quality of life. In this case report, we present a case of hyperthyroidism complicated by severe cholestatic jaundice and treated with methimazole. Through this case, we aim to provide insights and recommendations for managing such complex cases. A statement regarding obtaining informed consent from the patient for the publication of detailed information in this case report should be included in the case report.

## 2. Case presentation

A 43-year-old female patient was admitted to our department with a history of heat intolerance and excessive sweating for 13 years, along with recent onset of icterus and urine over the past week. She was diagnosed with hyperthyroidism 13 years back and was treated with methimazole, which led to symptom improvement. She had irregular follow-up visits and stopped her medication several times (the specific treatment process is unclear). One month before admission, the patient ceased her medication, subsequently experiencing palpitations and shortness of breath. She would become breathless after general physical activity or walking 100 meters, which could be relieved after rest. She also had edema in both lower limbs. One week before admission, she developed icteric sclera accompanied by dark urine, which gradually deepened to the color of tea, and her appetite decreased. Physical examination revealed a heart rate of 103 beats per minute, fatigue, protruding eyes, jaundice of the skin all over the body, no liver palms or spider nevi. The sclera was yellow, the thyroid was grade II enlarged, tough in texture, with a smooth surface, no small nodules were palpated, no tenderness, and no vascular murmurs were heard. No rales were heard in the lungs, the heart rhythm was regular, the abdomen was normal on examination, and there was pitting edema in both lower limbs. Initial laboratory tests showed thyroid function: free triiodothyronine 19.36 ng/L (normal value 2.14~4.2 ng/L), free thyroxine (FT4) 5.95 ng/dL (normal value 0.59~1.25 ng/dL), thyroid stimulating hormone 0.0008 uIU/mL (normal value 0.34~5.6 uIU/mL); anti-thyroglobulin antibody (TRAb) 30.85 KIU/L (normal value 0~115 KIU/L), thyroid peroxidase antibody 239.5 KIU/L (normal value 0~34 KIU/L). Routine blood test: white blood cells 6.30 × 10^9/L (normal value 3.5~9.5 × 10^9/L), hemoglobin 113g/L (normal value 115~150 g/L), platelets 130 × 10^9/L (normal value 125~350 × 10^9/L); liver function test: albumin 28.4g/L (normal value 40~55 g/L), total bilirubin (TBil) 333.2 umol/L (normal value ≤23 umol/L), direct bilirubin 181.0 umol/L (normal value ≤4 umol/L), indirect bilirubin 152.2umol/L (normal value ≤23 umol/L), aspartate aminotransferase (AST) 81U/L (normal value 13~35 U/L), alanine aminotransferase (ALT) 35U/L (normal value 7~10 U/L), alkaline phosphatase 265 U/L (normal value 35~100U/L). Coagulation function: prothrombin time 23.5S, international normalized ratio 2.07, partial thromboplastin time 36S, D-dimer measurement 0.57 mg/L FEU. B-type natriuretic peptide measurement 968 pg/mL (normal value 0~100 pg/mL). The patient serology for hepatitis A, B, C, D, and E were all negative, as were antibodies for autoimmune liver disease. Tumor markers (Alpha-fetoprotein, Carcinoembryonic antigen, CA199, CA125, CA153) were all negative. EB virus and cytomegalovirus IgM were both negative, ceruloplasmin 0.5 g/L (normal value 0.2~0.6 g/L). Thyroid ultrasound suggested thyroid enlargement, diffuse changes in the substance, and the possibility of hyperthyroidism. Echocardiogram: ejection fraction EF 74%: enlargement of the left atrium and right heart; widening of the pulmonary artery; tricuspid regurgitation (large amount); decreased left ventricular diastolic function (grade III); pulmonary hypertension (severe). Abdominal ultrasound suggested increased liver echo, abdominal effusion.

**Figure 1.**
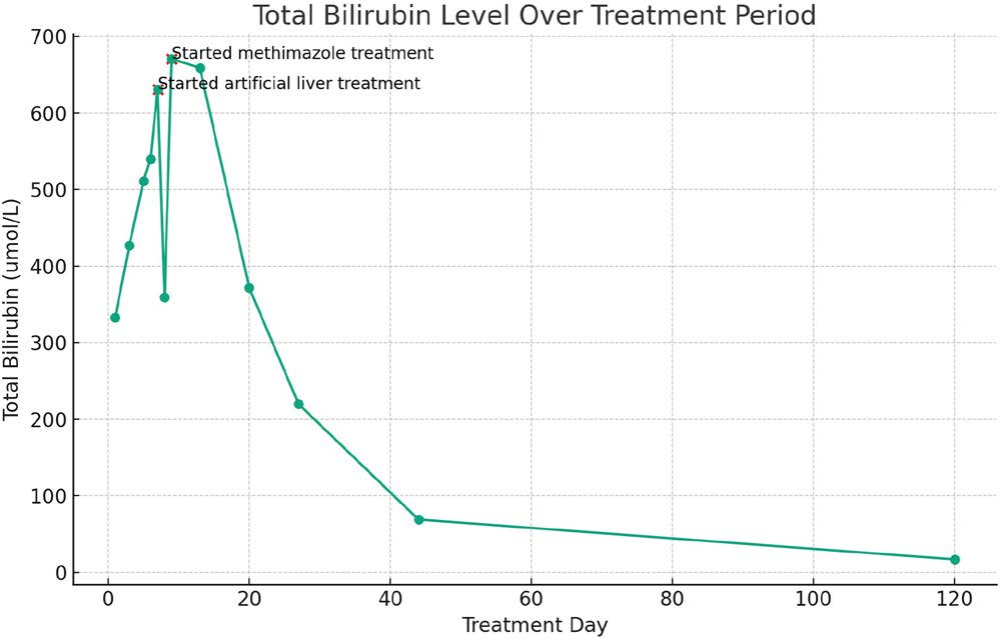
Total bilirubin level over treatment period.

**Figure 2.**
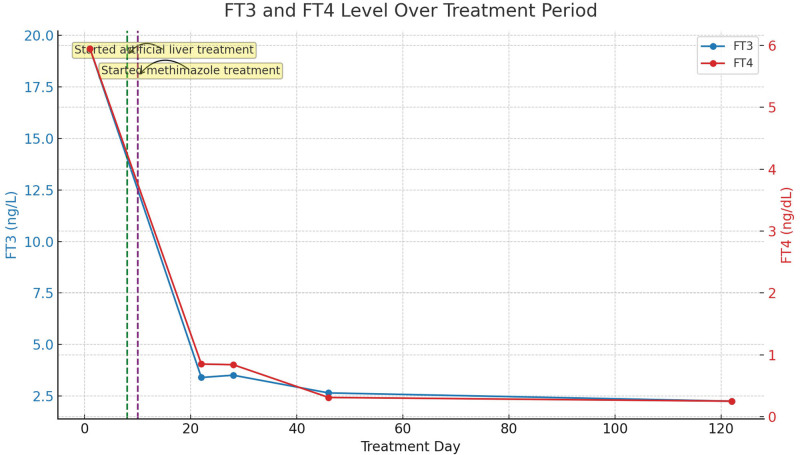
Free triiodothyronine (FT3) and free thyroxine (FT4) level over treatment period.

**Figure 3.**
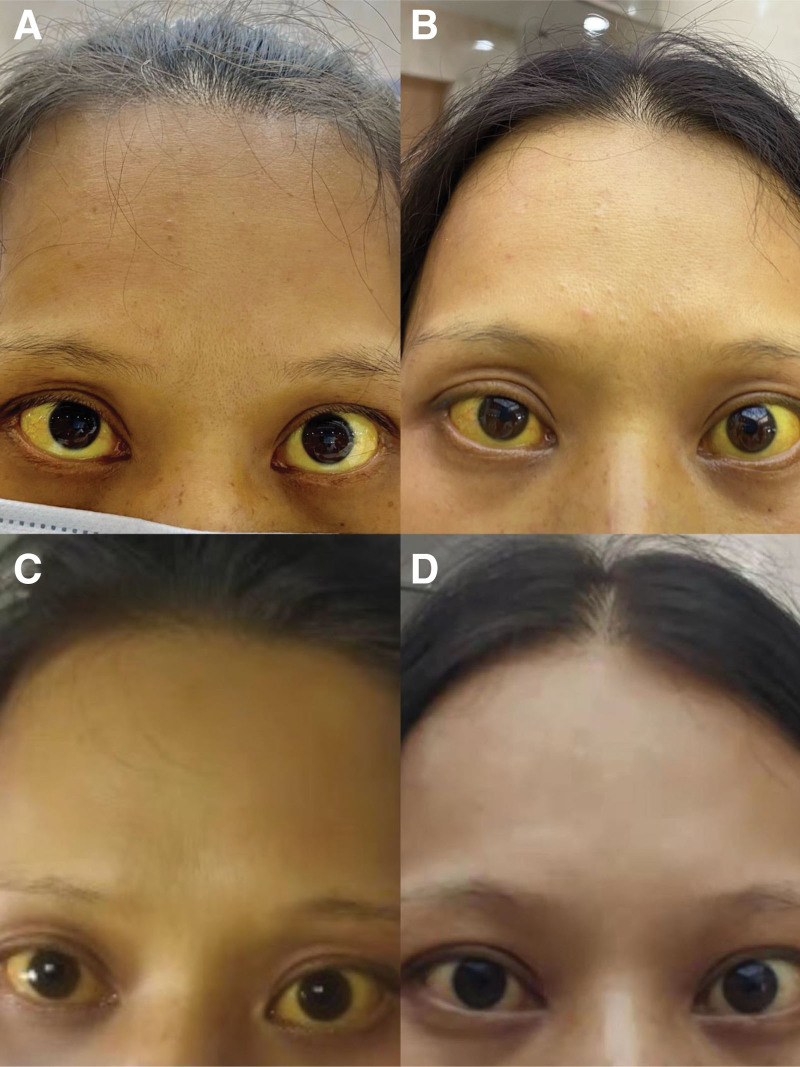
The patient exhibited changes in scleral icterus during the course of treatment. (A) Illustrates the patient scleral icterus on the 5th d of hospitalization. (B) Represents the patient scleral icterus on the 14th d of hospitalization. (C) Shows the scleral icterus 1 wk after discharge. (D) Depicts the scleral icterus 1 mo after discharge.

After excluding viral hepatitis, drug-induced, alcoholic, autoimmune, genetic metabolic and other liver diseases, the diagnosis was: Hyperthyroidism; Hyperthyroidism hepatic failure; Hyperthyroidism heart disease. After admission, the patient was given supportive treatment such as transfusion of fresh plasma, albumin, supplementation of vitamins, anti-inflammatory treatment with methylprednisolone, and treatment with compound glycyrrhizic acid, adenosylmethionine for liver protection, jaundice reduction and furosemide for diuresis. However, the condition did not improve significantly, the patient liver failure worsened, and the condition deteriorated (Fig. 1). On the 8th day of hospitalization, we had a full discussion with the patient family and added artificial liver support treatment once based on the original treatment. The review of liver function was better than before, showing albumin 34.5 g/L, TBil 359.6 umol/L, direct bilirubin 198.0 umol/L, indirect bilirubin 161.6 umol/L, AST 38U/L, ALT 23 U/L, but due to the patient financial problems, she refused to continue artificial liver treatment, and methimazole 10 mg bid was added orally on the 10th day of hospitalization. The patient coagulation function improved significantly, and the review of liver function on the 14th day of hospitalization was worse than before (Fig. 1). The patient refused to continue inpatient treatment and was discharged on her own. After discharge, the patient continued to take methimazole 10mg bid orally, compound glycyrrhizic acid for liver protection, and anti-inflammatory treatment with methylprednisolone. One week after discharge, the review of liver function suggested that bilirubin had significantly decreased compared to before. Three months after discharge, the patient was in good mental state, regular heart rhythm, normal liver function, coagulation function, thyroid function and other indicators (Figs. 1–3).

## 3. Discussion

Hyperthyroidism is a common endocrine system disease, which is caused by excessive thyroid hormones in the circulation due to various diseases of the thyroid itself or outside the thyroid, leading to increased excitability and metabolism of various systems such as nerves, circulation, and digestion. The liver is the main organ of thyroid hormone metabolism, so hyperthyroidism liver injury is common in clinical practice. The incidence of hyperthyroidism liver injury reported in the literature is 40% to 89%, and the liver injury is mostly mild to moderate, and hyperthyroidism combined with severe jaundice is rarely reported.^[3]^ Therefore, this case report focuses on the clinical characteristics and treatment strategy selection of hyperthyroidism combined with severe jaundice. The mechanism of hyperthyroidism combined with liver injury is not yet clear, possible reasons include: During hyperthyroidism, excessive thyroid toxins cause all organs in the body, including the liver, to be in a high metabolic state. Patients may show tachycardia, easy hunger, eating more, and weight loss. Currently, although the cardiac output is increased, the blood supply to the liver is not correspondingly increased, which leads to the liver being in a relatively ischemic and hypoxic state, causing liver injury.^[4]^ Some scholars have proposed a hypothesis for the mechanism of hyperthyroidism combined with severe jaundice, that hypoxia in the central area of the liver lobules may interfere with bile transport, leading to cholestasis and jaundice, but this hypothesis has not been confirmed.^[5]^ Severe hyperthyroidism may cause right heart failure, further exacerbating the state of liver ischemia and hypoxia and liver injury.^[6]^ During the treatment of hyperthyroidism, the use of antithyroid drug treatment (ATD) may also cause drug-induced liver injury, but the incidence is low. The literature reports that the incidence of ATD-related drug-induced liver injury is <0.5%, and most occur within the first 3 months of medication.^[7,8]^ Yang found that the probability of liver injury at the 4th, 8th, and 12th weeks of medication was 63.3%, 75.6%, and 81.1%, respectively.^[9]^ This patient has a history of intermittent oral methimazole for 13 years and has stopped the medication for more than 1 month, so drug-induced liver injury is temporarily not considered. Some studies have shown that hyperthyroidism liver injury may be related to the presence of high-titer TRAb and other autoantibodies in the body, and there is a strong autoimmune response.^[10]^ In general, hyperthyroidism can cause liver injury through multiple pathways, but the exact mechanism is not yet clear.

Early identification of hyperthyroidism high-risk groups that may be complicated by liver injury is of great significance, which can reduce the risk of severe jaundice, liver failure, death, etc. Zhang and others monitored the levels of phosphorus and cysteine S-glutathione transferase (CSHI) in serum, liver, and kidney, and found that the levels of phosphate and CSHI in serum were negatively correlated with the degree of hyperthyroidism liver injury, and the changes in phosphate and CSHI levels in liver and kidney tissues were positively correlated with the degree of hyperthyroidism liver injury, so they proposed that early detection of phosphate and CSHI levels in serum can effectively evaluate hyperthyroidism liver injury.^[11]^ But using thyroid function, thyroid antibody and other indicators to identify high-risk groups of hyperthyroidism liver injury is more worthy of clinical doctors reference. The literature shows that FT4 >75 pmol/L and TRAb >15 IU/L are independent predictors of hyperthyroidism liver injury,^[12]^ reminding clinical doctors to pay attention to the possibility of liver injury in this group of hyperthyroidism. Another study found that age >45 years, heart rate >90 beats/minute, thyroid mass >35 g, course of hyperthyroidism >3 years, FT4 ≥70.5 pmol/L, thyroid peroxidase antibody >360 IU/mL, and TRAb >15 IU/L are all risk factors for increasing hyperthyroidism liver injury, further narrowing the scope of the high-risk group of hyperthyroidism liver injury.^[13]^

The clinical manifestations of hyperthyroidism liver injury are varied, and the 3 most common types are cholestasis type, hepatocellular injury type, and liver synthesis dysfunction type. The liver injury is mostly mild to moderate, and severe liver injury is rare. Wafa et al summarized and summarized the clinical data of 17 newly diagnosed and untreated patients with hyperthyroidism combined with liver injury, and found that the types of liver injury included cholestasis (88.2%), hepatocellular injury type (41.2%) and liver synthesis dysfunction type (29.4%), while the incidence of severe liver injury was 11.8%, and the incidence of mild to moderate liver injury was 88.2%.^[14]^ Wang et al observed 2385 cases of Graves disease-related hyperthyroidism and found that the incidence of cholestasis type liver injury was 32.4%, while the incidence of severe liver injury was only 6.6%, among which severe liver injury was defined as: ALT or AST ≥ 20 × upper limit of normal, gamma-glutamyl transferase ≥ 10 × upper limit of normal, alkaline phosphatase ≥ 5 × ULN, and/or TBil ≥ 5 × ULN.^[15]^ At present, there are few comparative studies on severe liver injury and mild to moderate liver injury. This patient also mainly manifested as cholestasis type liver injury.

For the treatment of hyperthyroidism combined with severe jaundice, in addition to the conventional use of liver protection, jaundice reduction and other drugs, early and active control of hyperthyroidism is crucial to improve jaundice. However, hyperthyroidism and jaundice interact and restrain each other, and clinical treatment presents great challenges. The treatment plan for hyperthyroidism often includes ATD, iodine treatment and surgery. ATD itself has the risk of inducing drug-induced liver injury. Research has found that propylthiouracil (PTU)-induced liver injury is mainly characterized by varying degrees of hepatocyte necrosis, while MMI is more likely to show intrahepatic cholestasis, both of which can manifest as increased bilirubin or transaminase, but the incidence of ATD-related drug-induced liver injury is low, ranging from 0.1% to 0.2%.^[9]^ Wang and others observed 71,379 cases of ATD initially treated with hyperthyroidism and found that MMI has a higher risk of liver injury than PTU (3.17‰ vs 1.19‰), and a lower risk of acute liver failure (0.32‰ vs 0.68‰). The risk of MMI-induced liver injury is 2.89 times that of PTU, and the risk of large-dose MMI-induced liver injury is 5.08 times that of PTU.^[16]^ At present, the risk factors for ATD-related drug-induced liver injury are not clear, and some literature reports that old age and larger drug doses may be risk factors for ATD-related drug-induced liver injury.^[14]^ Therefore, clinical doctors often face the risk of aggravating liver injury in patients with hyperthyroidism and severe jaundice. Although these reports make us hesitate to use MMI to treat patients with cholestatic Graves disease, we believe that the severe cholestasis in this patient is caused by hyperthyroidism, not drug-induced liver injury caused by methimazole, and the patient refuses to continue artificial liver treatment due to financial difficulties, so the use of MMI to treat this patient is relatively safe and economical, and follow-up observation finally confirms the correctness of our clinical decision.

Limitations to Consider in This Case Report. Single Case: Results may not apply broadly to hyperthyroidism or liver dysfunction cases. Incomplete Data: Irregular follow-ups and medication lapses limit the study comprehensiveness.

In conclusion, when screening for the cause of liver injury, consider hyperthyroidism, and hyperthyroidism patients should be alert to the combination of severe jaundice, especially for hyperthyroidism patients with obvious hypermetabolic manifestations, high-titer thyroid antibodies, and involvement of organs outside the thyroid, more attention should be paid to closely monitoring liver function, coagulation function, etc. The incidence of hyperthyroidism combined with severe jaundice and liver failure is low, and the treatment of such patients is difficult and complex. In addition to symptomatic treatment such as liver protection and jaundice reduction, improving hyperthyroidism is crucial. ATD can be tried, pay attention to starting with a small dose, closely observe changes in liver function, and achieve the goal of successful rescue.

## Author contributions

**Conceptualization:** Boming Xu.

**Investigation:** Yilin Zeng.

**Supervision:** Peizhong Chen.

**Writing – original draft:** Xiaoqiang Liu.

**Writing – review & editing:** Yubin Wang.
